# The Book World of Medicine and Science

**Published:** 1904-05-28

**Authors:** 


### The Book World of Medicine and Science.

The Case against Anti-vivisection. By Stephen Paget. (London: The Scientific Press, Limited. Crown 8vo. 1904. Pp. 104. Price 2s. net.)

What We Owe to Experiments on Animals. By Stephen Paget. (London: The Scientific Press, Limited. Crown 8vo. 1904. Pp. 72. Price 1s. 6d. net.)

These two pamphlets fulfil admirably the object with which they were written. They present to the public interested in the advance of scientific medicine the results attained by research. They afford reasons for looking upon the anti-vivisection movement as a merely transient ebullition of no greater importance than the various anarchical propaganda, troublesome indeed whilst they exist, but destined to have no continuance of growth. "The Case against Anti-vivisection" shows that the various societies so far from being leagued together for a common object are divided amongst themselves, and are waging an internecine struggle for subscriptions, donations, and legacies to spend on offices, salaries, press cuttings, and reprints at well as for the more legitimate objects of lectures and meetings. The value of the pamphlet, like its subject, is ephemeral.

The second pamphlet entitled "What We Owe to Experiments on Animals" is of much more permanent value, and is worthy of careful study. It explains in plain words the knowledge gained by the help of experiments on animals in physiology, pathology, and practice. Mr. Paget is a master of English and he has made his pamphlet readable as well as intelligible. The first division of his book deals with physiology and opens with the circulation of the blood, beginning with the experiments of Galen—born more than a century before the Christian era—and ending fitly enough with Marey, who died last month. From the circulation he goes on to the lacteals, to the epoch-making discoveries of Réaumur and the Abbé Spallanzani in digestion, to the wonderful demonstration by Claude Bernard of the glycogenic function of the liver, wonderful because it gave the death-blow to the conception that each organ had but one function and that the animal body was to be regarded as a bundle of organs, each with its appropriate function working independently of all the rest. Discoveries in connection with the pancreas, the growth of bone, and the nervous system occupy the rest of the section, and in his summary Mr. Paget forecasts that the work of physiology will in the future continue to exercise as profound an influence for good as it has done in the past on the progress both of medical and surgical treatment. The second section deals in the same admirable manner with pathology, therapeutics, and bacteriology. Without the knowledge derived from experiments upon animals it is difficult to know how bacteriology could have attained its present value. Rabies and diphtheria would still have been treated empirically and there is very little doubt that the case mortality of diphtheria would have remained at 29 to 30 per cent. as it was before the introduction of antitoxin treatment, instead of becoming reduced to its present rate of 12 01 per cent. The experiments which led up to these results were not always free from danger to the experimenter. Pasteur in his first experiments with rabies used the saliva of rabid animals for his inoculations. The rabid beast in one case was a huge bulldog, foaming at the mouth and howling in his cage. All attempts failed to induce the animal to bite and infect a rabbit. "But we *must*," said Pasteur, "inoculate the rabbits with the saliva." Accordingly a noose was made and thrown, the dog secured and dragged to the edge of the cage, and his jaws tied together. Choking with rage, the eyes bloodshot, and the body convulsed by violent spasms the animal was stretched on a table and kept motionless while Pasteur, leaning over his foaming head, sucked up into a narrow glass tube some drops of the saliva. It is thus that Mr. Paget resembles the "blandi doctores" in Horace—

> "Ut pueris olim dant crustula blandi
> Doctores, elementa velint ut discere prima."

He lessens the severity of his theme by apposite trifles. The third section of his little book deals with the Vivisection Act, and shows how it is worked and what is meant by the various certificates granted under its authority.

The Health Resort, and Journal of Spas and Sanatoria. (London Publicity Company. Price 6d. monthly.)

The May number of this magazine contains several interesting articles dealing more or less closely, from the patients' standpoint, with such places as Bath, Bruges, Carlsbad and Biarritz. We even hear of the attractions of golf in the Riviera; and there is a good description of Gries in South Tyrol, a little town not much known to the English, but which seems to possess many of the charms of Italy, and is probably suited for those who are tired of the leaden skies of England or who suffer from brain-fag. Sunshine, palm trees, olive groves, and possibly even roses in January are tempting enough when they can be had within thirty-three hours of London and enjoyed amid the fine scenery of the Tyrol and the Dolomites. From a medical point of view the article most interesting at the present moment is that by Dr. Charles Reinhardt on the construction of sanatoria for consumption. Dr. Reinhardt has devoted much time to this subject and is a well-known authority. He has shown that the cheapest and best buildings for the purpose are wooden huts intended for one or two patients only, and therefore the palatial constructions for the treatment of consumptives find no favour in his eyes. Upon the whole we agree with Dr. Reinhardt, at least for the majority of patients. The magazine is well illustrated and printed.

Life and Work of James Compton Burnett, M.D. With an Account of the Burnett Memorial. Compiled by Dr. J. H. Clarke. (London: Published for the Burnett Memorial Committee, by the Homœopathic Publishing Company, 12 Warwick Lane, Paternoster Row, E.C. 1904. Price 2s. 6d. net.)

This is a biography of an eminent homœopath, written by an admiring friend. It will not be very helpful to outsiders. The reason why Dr. Burnett became an homœopath is stated by himself, in rather florid language, to have been the good results he obtained from the administration of aconite in the slight fevers which prelude so many diseases. This hardly seems to be a convincing explanation, but this little book gives no more scientific one. It does, however, make it clear that Dr. Burnett was a practitioner of considerable independence of thought, as well as abounding energy of expression. He claimed to have discovered and employed the method of treating tuberculosis by injections of its own virus, five years before Koch published his ideas. He also believed in the curability of tumours by means of medicines, a method which, however, he admits must be a slow one. He reckoned the average length of treatment as two years. The biography will doubtless please Dr. Burnett's friends and admirers, but it would have been more generally interesting and useful if Dr. Clarke had indulged in less effusion and more scientific lucidity.

